# Leader instigated task conflict and its effects on employee job crafting; the mediating role of employee attributions

**DOI:** 10.1371/journal.pone.0278329

**Published:** 2022-12-20

**Authors:** Ramsha Zakariya, Sayyed Muhammad Mehdi Raza Naqvi

**Affiliations:** Department of Management and Social Sciences, Capital University of Science and Technology, Islamabad, Pakistan; Universiti Pertahanan Nasional Malaysia, MALAYSIA

## Abstract

The role of leaders in conflict management remains the favorite area of researchers. This study aims to introduce a unique role of leaders in conflict named as leader instigated task conflict. We proposed that leader instigated task conflict promotes job crafting behaviors of employees by considering attributions regarding leader instigated conflict as mechanism for this relationship. Data were collected from 247 employees working in teams in marketing departments of different organizations across Pakistan. Data analyses was conducted through multilevel structural equation modeling. Findings revealed that leader instigated task conflict is positively related to promotion-focused job crafting and negatively related to prevention-focused job crafting via the mediating role of constructive conflict instigation attribution and destructive conflict instigation attribution respectively. The current study contributes to the literature on conflict by suggesting that leaders can be a source of task based conflict to achieve its positive outcomes. However, employee attributions play a vital role in achieving the positive outcomes of leader instigated task conflict, hence leaders must be careful about shaping constructive attributions of followers regarding their conflict instigation behavior in order to promote constructive behavioral responses of employees.

## Introduction

Over more than half a century, team conflict at work has continued to receive considerable attention of researchers due to its ubiquitous nature and its consequent effects on employee behaviors and performance [1–3]. Task conflict entails disagreements and difference of opinions regarding task activities, ideas, issues and content [4]. A stream of literature and evidence suggests its negative outcomes such as reduced satisfaction from job and diminished well-being [1, 5, 6]. However, a vast variety of literature emphasize that task related disagreements and debates are required at work in order to generate sharing of alternative ideas, constructive criticism, and debates regarding work tasks which promote positive work outcomes such as creativity and effective decision making [7, 8]. Hence, organizations look for suitable ways to promote sharing of conflicting ideas among team members.

Several factors have been identified which influence the process of conflict including individual and team related factors such as personality, collective experience, intragroup trust, team members’ approaches towards handling conflict and team climate among others [8–16]. Other studies have identified the role of leaders in managing team conflict. In this regard, leaders have been identified to occupy prominent role including conflict handling and conflict management roles which are largely studied in literature [17, 18].

However, since one of the primary functions of leadership is to provide direction to subordinates to achieve performance goals of their job [19], leaders can incite task related disagreements to direct team members for engaging in debates and discussions in order to achieve desirable behaviors at work [20]. This additional role of leaders in conflict is referred to as “leader’s conflict instigation” which is defined as leader’s behaviors that are involved in initiating or generating disagreements among group members [21]. Owing to its potential to extend insights related to task conflict as a potential factor to achieve desirable performance outcomes [21], the current study is an attempt to identify how leader instigated task conflict can result in desirable employee behaviors.

Additionally, attribution theory and attribution related research suggests that employees assign causal attributions to events or behaviors that they experience [22, 23]. These attributions result in behavioral reactions towards the events event or behavior thus observed or experienced. Similarly, employees develop attributions regarding their leader behaviors [23–27]. Hence, it is crucial to see what attributions followers ascribe to their leader’s conflict instigation behavior in order to see a clear picture of their behavioral response towards it [21]. This study is an attempt to study leader instigated conflict and its consequential conflict instigation attributions which trigger employees’ behavioral responses in the form of job crafting. Job crafting is characterized by employee behaviors that involve physical and cognitive changes which they make in their task or relational boundaries [28].

This study offers several theoretical and practical contributions to literature and practice. Firstly this research studies a unique role of leaders in team based task conflict by emphasizing on conflict instigation by leaders. In addition, the current study identifies employee attributions about leader instigated conflict to provide mechanism of how it takes a constructive route or follows a destructive route. Moreover, promotion focused and prevention focused job crafting behaviors as responses to conflict and its attribution are identified to propose additional behaviors other than the extensively studied behavioral responses to conflict [29–31]. Lastly, this study aims to provide insights to decision makers at organizations to look at leader’s role in conflict from a different perspective, proposing that task conflict promoted by leader ignites the spark necessary to engage team members in debates and discussions that can result in job crafting behaviors and hence improved ways of carrying out job.

## Hypothesis development

### Leader instigated task conflict and job crafting

Leader instigated task conflict is defined as the task related disagreements and debates among group members that are generated and promoted by leader [21]. Leaders’ instigate task related debates among group members in order to engage them in constructive debates. Previous studies suggest that several team activities require the participation of all team members. Activities where the ideas of all members are required, consensus and conformance rules among all members hinders the achievement of work goals [32]. Employees try to maintain their relational bonding with group members because of which they do not share their different opinions in order to develop agreement with fellow workers [33]. This impairs honest and intellectual discussion and debates among group members on different alternatives and leads to ineffective decision making [34, 35].

On these grounds, leaders may become a source of generation and discussion of conflicting ideas among team members in order to motivate group members to participate in debates and discussions and share their ideas for learning and growth of team members [21]. Hence, it may result in employee behaviors that involve sharing of knowledge which helps team members in bringing improvements to their tasks as well as suggesting better work procedures. This behavior characterized by bringing improvements in one’s job through growth and learning is termed as job crafting [28]. In this regard job crafting is distinguished into three types of behaviors namely increasing structural and social job resources, increasing challenges and decreasing job demands [36]. For example, an employee may increase social job resources by seeking advice from coworkers, increase structural job resources by learning new things at job, increase challenging job demands by taking extra tasks, or reduce hindering job demands by avoiding contact with unpleasant clients [37]. Literature further classifies job crafting as promotion focused and prevention focused behaviors [38]. Increasing resources and challenges is named as promotion focused job crafting while reducing demands is referred to as prevention focused job crafting [39].

We suggest that leaders instigate group task conflict to encourage group members to engage in debates and discussion of alternative viewpoints. This discussion, under the supervision of leader, boosts morale and confidence of followers to bring forth their ideas and opinions about devising improvements in work. Furthermore, this leader instigated task conflict also motivates team members to speak up about the issues and problems they face which may otherwise lead them to engage in self-undermining and avoidance behaviors. Hence leader instigated task conflict facilitates promotion-focused job crafting and diminishes prevention-focused job crafting.

***Hypothesis 1a***: *Leader instigated task conflict is positively associated with promotion-focused job crafting*.
***Hypothesis 1b***: *Leader instigated task conflict is negatively associated with prevention-focused job crafting*.

### Leader instigated task conflict and conflict instigation attributions

The response of employees to leader instigated conflict task depends upon their appraisal of leader’s motives for instigating the conflict. Attribution theory [40] suggests that individuals’ reactions to events are not only based on the event itself, rather they are based on the motives they assign to the actor for certain behaviors [41]. Recent studies have provided evidence that employees also assign causal attributions to the behavior of their leader [23, 42]. This suggests that employees are more likely to assign attributions to leader’s instigation of group conflict by categorizing it to be caused by constructive or destructive intentions. Hence, the response of employees towards leader instigated task conflict will be based on the attribution of conflict instigation. Studies on employee attributions towards leader’s behaviors generally conceptualize them on the basis of constructive or destructive motives of leaders [43]. Similarly, conflict instigation behavior of leaders is attributed by followers in two forms; constructive in order to provoke team members to share their ideas and facilitating information processing or destructive in order to cause hurt or injury and to criticize employees [21]. It is argued that when leaders generate and facilitate task conflict in team, they are making their followers engaged in task related debates and discussions that are supervised and guided by the leader. It happens because the leader creates environment where team members feel comfortable to share their ideas, alternatives and critical opinions which promotes debates among team members regarding task at hand. Hence leader generated task conflict as facilitated by the leader’s involvement is more likely to develop constructive attributions of the followers. Similarly, followers may not develop destructive conflict instigation attribution observing that leader is also involved in group level debates. Further, as team members observe that they are encouraged for sharing their alternative viewpoints and ideas and appreciated on discussion based problem solving and new solutions, they are not likely to develop destructive attributions for leader behaviors directed towards task related conflict instigation.

***Hypothesis 2a***: *Leader instigated task conflict is positively associated with constructive conflict instigation attribution*
***Hypothesis 2b***: *Leader instigated task conflict is negatively associated with destructive conflict instigation attribution*

### Conflict instigation attributions and job crafting

Job crafting involves suggesting and making positive changes in the job in order to bring work improvements and achieve job related goals [28]. Considering that leaders instigate task conflict in order to promote growth, learning and improvement of group members [21], it is argued that individuals who believe that conflict is instigated by leader to generate constructive debate in order to make the team members learn through healthy discussions among team members are more likely to respond with learning new things regarding their tasks. Under the influence of constructive conflict instigation attribution, they are more likely to respond through improving their aspects of job which with the purpose of advancement, growth and accomplishment [44]. Moreover, taking leader instigated conflict as an opportunity to develop and share new ideas, and also to maintain cooperative relationships with a focus on achievement of work goals, employees are more likely to bring changes to their job that they consider to be helpful. This will lead employees to adopt promotion oriented job crafting behaviors. On the other hand, destructive conflict instigation attribution results in avoidance and withdrawal behaviors whereby employees don’t see learning and improvement opportunities [21]. Hence they are more likely to try to adopt undermining behaviors such as trying to reduce or avoid taking up tasks and also limit their social resources in order to avoid interactions with other coworkers which leads to prevention oriented job crafting.

***Hypothesis 3a***: *Constructive conflict instigation attribution is positively related to promotion-focused job crafting*.
***Hypothesis 3b***: *Destructive conflict instigation attribution is positively related to prevention-focused job crafting*.

### Indirect effect of leader instigated task conflict on job crafting through conflict instigation attributions

Based on above literature and hypotheses 2 and 3, it is suggested that leader instigated task conflict promotes constructive conflict instigation attribution and reduces constructive conflict instigation attribution. The constructive attributions further facilitate growth, learning and improvement in job and hence result in promotion focused job crafting. On the other hand, task conflict instigation by leader reduces destructive attributions which may result in self-undermining behaviors termed as prevention-focused job crafting. This leads to the following hypotheses:

***Hypothesis 4a***: *Constructive conflict instigation attribution mediates the relation between leader instigated task conflict and promotion-focused job crafting*.
***Hypothesis 4b***: *Destructive conflict instigation attribution mediates the relation between leader instigated task conflict and prevention-focused job crafting*.

**Fig 1:**
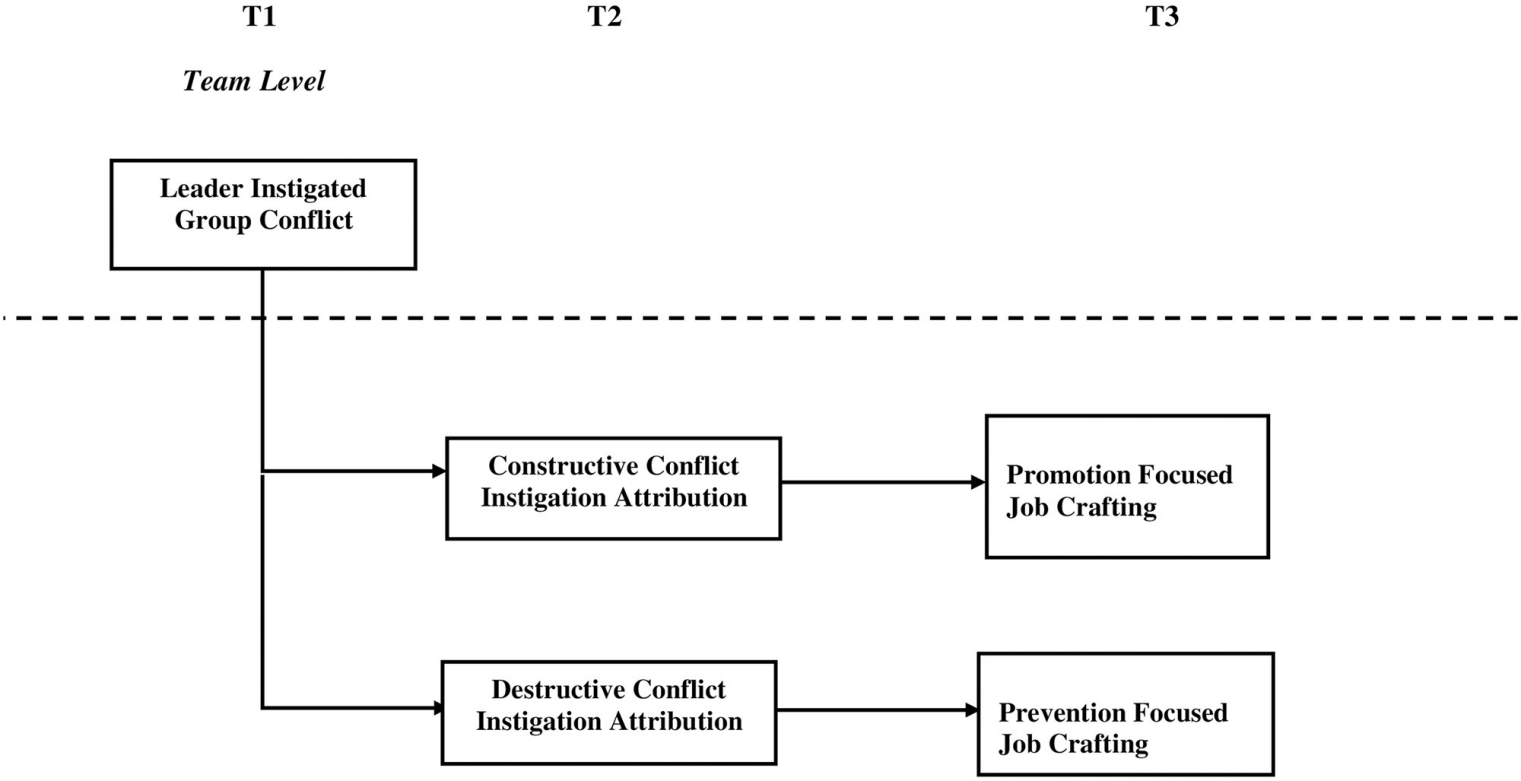
Research model. A multilevel model of leader instigated task conflict, attributions and job crafting.

The proposed research model for the hypothesized relations of leader instigated task conflict, attributions and job crafting is presented in Fig 1.

## Materials and methods

### Population, sample and procedure

Self-administered paper-based survey questionnaires were used to collect data. A number of previous studies used this method [45, 46]. Data collection through questionnaires is one of the most widely used methods because it provides data on standardized questions efficiently which can be used to obtain statistically useful findings that are generalizable for large populations [47]. The population of the present study comprises of full-time employees working in teams in the marketing and sales departments of public and private sector organizations of Pakistan.

The first author approached HR managers of eighteen public and private sector organizations of Pakistan on the basis of personal and professional contacts with the request to grant access to their marketing and sales teams to participate in our study. Sixteen organizations gave willingness to participate in the research project. The first author then approached employees of participating organizations and communicated the study purpose and distributed questionnaires to those employees who gave verbal consent to participate in the study. A cover letter highlighting the purpose of study and confidentiality of data and was handed to each employee along with the questionnaire. Participants were ensured that all the data they provide will remain confidential as the data analysis will be conducted at an aggregate level and findings will be generalized over the population of study. Further, they were informed that no data will be made public by any means thereby ruling out of confidentiality concerns.

Three survey forms (A-I, B-II, C-III) were used to collect the data in three points of time. At time-1 (t_1_), employees filled form A-I to report data about their demographics and task conflict instigation of their leader. An employee ID was assigned to each response collected in order to trace out the same employees for data collection process in different time lags. At time-2 (t_2_), employees who responded to survey form A-I were contacted through the assigned ID and survey forms B-II were distributed to report constructive and destructive conflict instigation attributions. At time-3 (t_3_), only those employees were contacted who responded to all survey items on survey forms including A-I and B-II and they were asked to respond to third survey form C-III to report their level of involvement in promotion and prevention job crafting. The maximum gap between each time lag was two weeks.

A total of 400 questionnaires were distributed out of which 264 responses were received back after all three time lags. 17 survey forms were not usable due to unmatchable response. After excluding incomplete responses, the final sample consisted of 247 employees embedded in 51 teams (response rate 61.75%) with a majority comprising of males (69.3%), 25–30 years of age (59.2%) and 3–6 years of job experience (54.4%).

Ethical approval for data collection was obtained from the Departmental Ethics Committee of Capital University of Science and Technology, Islamabad. The data collection procedure conformed to the ethical guidelines. Our study did not involve clinical trials or experiments with human beings or animals and the content of the questionnaires had no sensitive information requirement.

### Instrumentation

The survey questionnaire included three sections. The first section included a cover letter explaining the purpose of study along with statement of confidentiality and voluntary participation. The second section included demographic variables including team ID, employee ID, gender, age and experience. The third section included variables of the research model: leader instigated group conflict (measured at time-1), constructive and destructive conflict instigation attributions (measured at time-2), and job crafting (measured at time-3).

All items were measured on a five point Likert scale ranging from 1 (strongly disagree) to 5 (strongly agree). We adapted the refined version used by [48] of the Intragroup Conflict Scale [49, 50] to measure leader instigated group conflict. Sample item includes “My team leader invites my team members to discuss evidence for alternative viewpoints”. Constructive and destructive attributions were measured by adapting a scale developed by Liu and Liao [26] for measuring employee attributions for leader behavior. Sample items include “Desire to stimulate team members to meet performance goals” for constructive attribution and “Desire to hurt feelings of team members” for destructive attribution. Job crafting was measured with 21-itme scale developed by Tims and Bakker [37] and adapted and used by Lichtenthaler and Fischbach [39]. A sample item is: “I try to learn new things at work”.

## Analysis and results

### Multilevel data analysis strategy

The data analysis for this study was conducted through multilevel path analysis. The average cluster size (number of employee reporting to each team/department leader) was 4.84. Since leader instigated task conflict was conceptualized at group level, we followed the approach suggested by [51] and calculated average interrater agreement coefficient *r*_WG_ and intraclass correlation including ICC1 and ICC2 [52]. The *r*_WG_ had a mean of 0.89 and median of 0.93 (SD = 0.15) which meets the conventionally acceptable threshold of 0.70 [53] suggesting that the responses of group members within same group were consistent. The ICC1 value was 0.65 which meets the minimum threshold criteria of 0.10 suggesting that being in one group influences employee’s perceptions about leader instigated conflict. Moreover ICC2 was 0.84 which meets the minimum threshold criteria of 0.70 [51]. The theoretical arguments and ICCs for this study demonstrate the need to adopt multilevel path analysis approach [54].

### Measurement model

Confirmatory factor analysis were conducted on latent variables to test the measurement model in order to examine model fitness, factor structure and validity of the data. We used multiple indices criteria including relative chi-square χ^2^/DF (threshold > 3), Tucker-Lewis Index (threshold .90), comparative fit index (threshold .95), Root Mean Square Error of Approximation (threshold .08–.05) to examine model fitness [55, 56]. We conducted CFA with all five latent variables including leader instigated team conflict, constructive and destructive conflict instigation attribution, promotion and prevention focused job crafting which accounted for adequate model fitness (χ2/df = 2.47, CFI = 0.92, IFI = .93, TLI = .90, RMSEA = 0.05). We also accounted for the convergent and discriminant validity of study variables. The factor loadings for all indicators of latent variables ranged from 0.70 to 0.96 hence indicating convergent validity. In addition, discriminant validity was examined through comparing model fitness of alternative models with the hypothesized five-factor model. Table 1 shows that the hypothesized five-factor model fit the data better than all other alternative four-factor, three-factor and one factor models. Thus, the discriminant validity of all study variables is established.

**Table 1:** Confirmatory factor analysis.

Model	χ^2^/df	RMSEA	IFI	TLI	CFI
**Five-factor model**:	2.47	.05	.93	.90	.92
Hypothesized Model					
**Four-factor model 1**:	2.88	.07	.74	.72	.73
CCIA and DCIA combined					
**Four-factor model 2**:	2.96	.09	.61	.59	.60
Promotion JC and Prevention JC combined					
**Three-factor model**:	3.21	.12	.61	.59	.60
CCIA and DCIA combined and Promotion JC and Prevention JC combined					
**One-factor model**:	4.55	.19	.48	.42	.49
All variables combined into one factor					

CCIA = Constructive conflict instigation attribution, DCIA = Destructive conflict instigation attribution, Promotion JC = Promotion focused job crafting, Prevention JC = Prevention focused job crafting, RMSEA = Root Mean Square Error of Approximation, IFI = Incremental Fit Index, TLI = Tucker Lewis index, CFI = Comparative Fix Index

### Descriptive statistics

Table 2 presents means, standard deviations, reliability and correlations of the variables under study. The Chronbach’s alpha values for all variables were greater than 0.7. As shown in table, the correlations between study variables were in expected direction. Leader instigated task conflict was positively and significantly related to promotion focused job crafting (*r* = .541, *p* < .05) while it was negatively related to prevention focused job crafting (*r* = -.409, *p* < .05). Moreover, leader instigated task conflict was positively and significantly related to constructive conflict instigation attribution (*r* = .339, *p* < .05) while it was negatively related to destructive conflict instigation attribution (*r* = -.408, *p* < .05). Lastly, constructive conflict instigation attribution was positively associated with promotion focused job crafting (*r* = .449, *p* < .05) and destructive conflict instigation attribution was positively associated with prevention focused job crafting (*r* = .400, *p* < .05).

**Table 2:** Means, standard deviation and correlations variables.

	Means	SD	LITC	CA	DA	PRO_JC	PRE_JC
**LITC**	3.93	.847	(.849)				
**CCIA**	4.04	.561	.339**	(.829)			
**DCIA**	2.67	.495	-.408**	-.193**	(.837)		
**PRO_JC**	3.93	.668	.541**	.449**	-.340**	(.963)	
**PRE_JC**	2.74	.918	-.409**	-.156*	.400**	-.269**	(.942)

N = 247,

**p<0.01,

* p<0.05,

values in parenthesis indicate Cronbach alpha reliability. LITC = Leader instigated team conflict, CCIA = Constructive conflict instigation attribution, DCIA = Destructive conflict instigation attribution, PRO_JC = Promotion focused job crafting, PRE_JC = Prevention focused job crafting

### Hypothesis testing

Our proposed model included variables at multiple levels, with the independent variable leader instigated task conflict at level 2 while the mediators constructive and destructive conflict instigation attribution and dependent variables promotion and prevention focused job crafting were individual level constructs. We conducted multilevel analysis in Mplus (8.0) following the approach suggested by Preacher, Zhang [57]. For hypotheses testing, we considered bias corrected bootstrapping with 95% confidence interval along with significance [58].

The direct and indirect effects proposed in study hypotheses are shown in Table 3 which presents standardized path coefficients along with corresponding significance values. H1a and H1b proposed a positive relation of leader instigated task conflict and promotion focused job crafting while negative relation between leader instigated task conflict and prevention focused job crating respectively. Both these hypothesis are supported as leader instigated task conflict and promotion focused job crafting are positively related (β = .574, *p* < .05, LLCI = .308, ULCI = .840) while negative relation between leader instigated task conflict and prevention focused job crafting (β = -.531, *p* < .05, LLCI = -.761, ULCI = -.303). H2a and H2b proposed that leader instigated task conflict is positively related to constructive conflict instigation attribution while it is negatively related to destructive conflict instigation attribution. A positive relation was found between leader instigated task conflict and constructive conflict instigation attribution (β = .464, *p* < .05, LLCI = .177, ULCI = .750) while a negative relationship was found between leader instigated task conflict and destructive conflict instigation attribution (β = -.484, *p* < .05, LLCI = -.765, ULCI = -.213) thereby accepting H2a and H2b. Furthermore, H3a and H3b predicted positive relations between constructive conflict instigation attribution and promotion focused job crafting, and destructive conflict instigation attribution and prevention focused job crafting respectively. These relations are found consistent to hypothesized directions with a positive significant relation between constructive conflict instigation attribution and promotion focused job crafting (β = .530, *p* < .05, LLCI = .264, ULCI = .795), and destructive conflict instigation attribution and prevention focused job crafting (β = .668, *p* < .05, LLCI = .411, ULCI = .925) thereby accepting H3a and H3b.

**Table 3:** Path coefficients in the baseline model.

Relationship	Coefficients	95% CI	
		LLCI	ULCI
***Direct Effects***			
LITC ➝ Promotion JC	.574***	.308	.840
LITC ➝ Prevention JC	-.531***	-.761	-.303
LITC ➝ CCIA	.464**	.177	.750
LITC ➝ DCIA	-.484**	-.765	-.213
CCIA ➝ Promotion JC	.530***	.264	.795
DCIA ➝ Prevention JC	.668***	.411	.925
***Indirect Effects***			
LITC ➝ CCIA ➝ Promotion JC	.246**	.026	.466
LITC ➝ DCIA ➝ Prevention JC	-.324**	-.555	-.092

***p<0.001,

**p<0.01,

LITC = Leader instigated team conflict, CCIA = Constructive conflict instigation attribution, DCIA = Destructive conflict instigation attribution, Promotion JC = Promotion focused job crafting, Prevention JC = Prevention focused job crafting

Lastly, H4a and H4b proposed the indirect effect of leader instigated task conflict on promotion focused job crafting and prevention focused job crafting via the mediation of constructive conflict instigation attribution and destructive conflict instigation attribution respectively. We accounted for indirect effect through bootstrapping with bias corrected 95% confidence interval. It was found that the upper limit and lower limit confidence interval of the true indirect effect of leader instigated task conflict on promotion focused job crafting did not contain zero therefore the indirect effect is significant (β = .246, *p* < .05, LLCI = .026, ULCI = .466). Similarly the indirect effect of leader instigated task conflict on prevention focused job crafting is significantly different from zero as the upper limit and lower limit confidence intervals do not contain zero (β = -.324, *p* < .05, LLCI = -.555, ULCI = -.092). Hence H4a and H4b are accepted.

In summary, H1a and H1b proposed a positive relation of leader instigated task conflict and promotion focused job crafting while negative relation between leader instigated task conflict and prevention focused job crating respectively. As described above, both of the hypothesis are accepted. Hypothesis H2a and H2b are also accepted showing that leader instigated task conflict is positively related to constructive conflict instigation attribution while it is negatively related to destructive conflict instigation attribution. Additionally, results show that constructive conflict instigation attribution mediates between leader instigated task conflict on promotion focused job crafting hence H4a is also accepted. Finally, destructive conflict instigation attribution is found to mediate between leader instigated task conflict and prevention focused job crafting therefore H4b is also accepted.

## Discussion

The purpose of this study was to introduce an additional role of leaders in conflict, namely leader instigated task conflict at group level and examine its behavioral outcomes at workplace. Furthermore, we examined whether this relationship is mediated by employee attributions regarding leader conflict instigation behavior. Our study findings indicate that leader instigated task conflict leads to job crafting behaviors of employees. More specifically, we found that leader behaviors of inciting task conflict in their group facilitate promotion-focused job crafting behaviors. This finding is supported by previous studies which suggest that leader’s behaviors provide direction to followers regarding their behaviors at work [59]. This finding is also supported by mainstream literature of conflict which suggests that engaging in task related disagreements at work bring positive effects to work outcomes [20, 60]. Further, in line with previous literature which suggests that organizations should promote task conflict among teams in order to achieve effective performance aimed at developing creative solutions [7, 8, 61–63], we also suggest that managers who want their employees to improve their work mechanisms should promote and appreciate task related conflict. Our results additionally suggest that task related conflict promoted by leader can bring creative ideas, solutions and improved work approaches.

Further, we found significant role of attributions in shaping employees’ responses towards leader instigated task conflict. This finding is supported by previous literature suggesting that attributions of employees regarding their leader’s behaviors shape their behavioral responses [24, 42]. We highlight that role of leaders in this phenomenon is imperative since their behavior can shape employee attributions to be constructive or destructive. Leaders instigated task conflict that is effectively supervised by the leader has more prospects of yielding constructive attribution. Leaders who are able to tolerate contradictions and display their encouragement of bringing up different ideas are particularly good at instigating constructive attributions of followers. Therefore, leaders with constructive intentions behind their conflict instigation behaviors are focused on achieving improved performance standards and targets [21]. Hence displays of such behaviors by leaders is suggested in order to foster constructive attributions of employees.

Lastly, the findings suggest that leaders are required to keep careful consideration that destructive attribution can lead to more destructive route resulting in self-undermining and avoidance behaviors namely prevention-focused job crafting which can be detrimental for performance of employees. Employees are more likely to develop destructive attributions if leader behavior is derogatory, manipulative or hurting [21]. Hence, leaders must focus on shaping positive attributions of followers by clearly communicating their motives of conflict instigation aimed at improved performance and achievement of performance goals instead of causing damage or hurt to any team member.

### Limitations and future directions

Despite several strengths, the current study is not without limitations. Although we collected data in three time intervals from employees in order to reduce the likelihood of single source data, our study cannot be considered a longitudinal research design. We suggest future studies to replicate our research model by using a pure longitudinal research design in order to rule out biases associated with cross sectional research design. The current study conceptualizes leader instigated conflict with respondents being the team members and not the leader, there can be difference of opinion between leader regarding his/her conflict instigation behavior and employee’s construal of this phenomenon. This phenomenon can be recorded from the lens of leader in future studies, however, since the aim of current study was focused on employees’ behavioral response towards leader’s conflict instigation behavior, it was necessary to look at this phenomenon from employees’ perspective since individuals response to their perception of actor’s behaviors and not what the actors perceive about their own behavior. Future studies can also aim their research questions around looking at leader instigated conflict from leader’s perspective as well as follower’s perspective and compare and contrast the differences in both phenomena. We believe that findings of this study will equally benefit all kinds of jobs where achievement of performance goals is subject to debates and discussion among team members such as R&D and strategic management. Such debates are likely to promote desired improvement in behaviors of employees where they craft their jobs for better results. However, more studies are required to validate our findings in different organizations, hence we invite future researchers to test our proposed model in professional settings that require discussion and input from team members.

It is also important to inquire if the same findings will prevail in careers other than marketing such as finance and accounts, operations, administration and HR management among others. Nonetheless, one might anticipate that findings may revert in bureaucratic work settings that follow strict SOPs. Hence, it opens room for further research to be carried out in this domain in multiple work settings. Lastly, the likelihood exists that a number of conditional factors may shape the attributions of employees regarding leader’s conflict instigation. Future researchers can identify constructs that can act as conditional factors in this association with the help of attribution theory [40] and attribution related research. Other conditional factors can be identified by drawing on previous literature that have identified factors that shape the process and outcomes of conflict.

## Supporting information

S1 File (SAV)

S2 File (DOCX)
