# Supplementary material for: Leader instigated task conflict and its effects on employee job crafting; the mediating role of employee attributions
Source: PLoS One. 2022 Dec 20;17(12):e0278329. doi: 10.1371/journal.pone.0278329 (PMC9767343; doi:10.1371/journal.pone.0278329)
Supplement: S2 File — (DOCX) [file pone.0278329.s002.docx]

| **LEADER INSTIGATED GROUP CONFLICT** |
| --- |

**Rate the following statements, while keeping in view your *IMMEDIATE SUPERVISOR*.**

Response Key: 1 = Strongly Disagree, 2 = Disagree, 3 = Neutral, 4 = Agree, 5 = Strongly Agree

|  | *My team leader incites/invites:* |  |  |  |  |  |
| --- | --- | --- | --- | --- | --- | --- |
| 1 | My team members to argue the pros and cons of different opinions | 1 | 2 | 3 | 4 | 5 |
| 2 | My team members to discuss evidence for alternative viewpoints | 1 | 2 | 3 | 4 | 5 |
| 3 | My team members to engage in debate about different opinions or ideas | 1 | 2 | 3 | 4 | 5 |
|  |  |  |  |  |  |  |

| **CONFLICT INSTIGATION ATTRIBUTION** |
| --- |

Response Key: 1 = Strongly Disagree, 2 = Disagree, 3 = Neutral, 4 = Agree, 5 = Strongly Agree

*Task conflict has been defined as “disagreements among group members about the content of the tasks being performed, including differences in viewpoints, ideas, and opinions”. To what extent, do you agree that the following may be the reason for or cause of your leader’s task conflict development in your group?*

|  | Constructive attribution | | | | | |
| --- | --- | --- | --- | --- | --- | --- |
| 1 | Desire to elicit high performance from me | 1 | 2 | 3 | 4 | 5 |
| 2 | Desire to stimulate me to share my ideas | 1 | 2 | 3 | 4 | 5 |
| 3 | Desire to push me to work harder | 1 | 2 | 3 | 4 | 5 |
| 4 | Desire to push me to come out of comfort zone. | 1 | 2 | 3 | 4 | 5 |
| 5 | Desire to stimulate me to meet my performance goals. | 1 | 2 | 3 | 4 | 5 |

|  | Destructive attribution | | | | | |
| --- | --- | --- | --- | --- | --- | --- |
| 1 | Desire to cause injury on me | 1 | 2 | 3 | 4 | 5 |
| 2 | Desire to hurt my feelings | 1 | 2 | 3 | 4 | 5 |
| 3 | Desire to cause tension between team members | 1 | 2 | 3 | 4 | 5 |
| 4 | Desire to make me feel bad about myself | 1 | 2 | 3 | 4 | 5 |
| 5 | Desire to retaliate me | 1 | 2 | 3 | 4 | 5 |

| **JOB CRAFTING** |
| --- |

**Indicate the extent of your agreement or disagreement with the below statements**

Response Key: 1 = Strongly Disagree, 2 = Disagree, 3 = Neutral, 4 = Agree, 5 = Strongly Agree

|  | Increasing structural job resources |  |  |  |  |  |
| --- | --- | --- | --- | --- | --- | --- |
|  | I try to develop my capabilities | 1 | 2 | 3 | 4 | 5 |
|  | I try to develop myself professionally | 1 | 2 | 3 | 4 | 5 |
|  | I try to learn new things at work | 1 | 2 | 3 | 4 | 5 |
|  | I make sure that I use my capacities to the fullest | 1 | 2 | 3 | 4 | 5 |
|  | I decide on my own how I do things | 1 | 2 | 3 | 4 | 5 |
|  | Decreasing hindering job demands |  |  |  |  |  |
|  | I make sure that my work is mentally less intense | 1 | 2 | 3 | 4 | 5 |
|  | I try to ensure that my work is emotionally less intense | 1 | 2 | 3 | 4 | 5 |
|  | I manage my work so that I try to minimize contact with people whose problems affect me emotionally | 1 | 2 | 3 | 4 | 5 |
|  | I organize my work so as to minimize contact with people whose expectations are unrealistic | 1 | 2 | 3 | 4 | 5 |
|  | I try to ensure that I do not have to make many difficult decisions at work | 1 | 2 | 3 | 4 | 5 |
|  | I organize my work in such a way to make sure that I do not have to concentrate for too long a period at once | 1 | 2 | 3 | 4 | 5 |
|  | Increasing social job resources |  |  |  |  |  |
|  | I ask my supervisor to coach me | 1 | 2 | 3 | 4 | 5 |
|  | I ask whether my supervisor is satisfied with my work | 1 | 2 | 3 | 4 | 5 |
|  | I look to my supervisor for inspiration | 1 | 2 | 3 | 4 | 5 |
|  | I ask others for feedback on my job performance | 1 | 2 | 3 | 4 | 5 |
|  | I ask colleagues for advice | 1 | 2 | 3 | 4 | 5 |
|  | Increasing challenging job demands |  |  |  |  |  |
|  | When an interesting project comes along, I offer myself proactively as project co-worker | 1 | 2 | 3 | 4 | 5 |
|  | If there are new developments, I am one of the first to learn about them and try them out | 1 | 2 | 3 | 4 | 5 |
|  | When there is not much to do at work, I see it as a chance to start new projects | 1 | 2 | 3 | 4 | 5 |
|  | I regularly take on extra tasks even though I do not receive extra salary for them | 1 | 2 | 3 | 4 | 5 |
|  | I try to make my work more challenging by examining the underlying relationships between aspects of my job | 1 | 2 | 3 | 4 | 5 |
